# Discrete Particle Swarm Optimization with Scout Particles for Library Materials Acquisition

**DOI:** 10.1155/2013/636484

**Published:** 2013-09-01

**Authors:** Yi-Ling Wu, Tsu-Feng Ho, Shyong Jian Shyu, Bertrand M. T. Lin

**Affiliations:** ^1^Institute of Information Management, National Chiao Tung University, Hsinchu 30010, Taiwan; ^2^Department of Computer Science and Information Engineering, Ming Chuan University, Taoyuan 33348, Taiwan

## Abstract

Materials acquisition is one of the critical challenges faced by academic libraries. This paper presents an integer programming model of the studied problem by considering how to select materials in order to maximize the average preference and the budget execution rate under some practical restrictions including departmental budget, limitation of the number of materials in each category and each language. To tackle the constrained problem, we propose a discrete particle swarm optimization (DPSO) with scout particles, where each particle, represented as a binary matrix, corresponds to a candidate solution to the problem. An initialization algorithm and a penalty function are designed to cope with the constraints, and the scout particles are employed to enhance the exploration within the solution space. To demonstrate the effectiveness and efficiency of the proposed DPSO, a series of computational experiments are designed and conducted. The results are statistically analyzed, and it is evinced that the proposed DPSO is an effective approach for the studied problem.

## 1. Introduction

In recent years, the price inflation of library materials, the shrinking of library budget, and the growth of electronic resources continue to challenge the acquisition librarians [1]. Complicating the effects of these challenges is the growth of scholarly and popular publications. With the great increase in publications, the librarians have not only to acquire the latest and the preferred materials within the limited budget but also to take the collection policy into consideration. Walters [2] reports that the annual inflation rate of academic books and periodicals were 1.4 and 8.5 percent. The research planning and review committee of the Association of College and Research Libraries (ACRL) [3] develops the 2010 top ten trends in academic libraries and finds that many libraries will face the budget pressure in the near future. These reaffirm the fact that the materials acquisition problem is exacerbated by the difficulty of aligning the library offerings with patron needs under the budget pressure. 

Over the past few decades, researches on materials acquisition have been conducted and implemented with a number of operations research based models and approaches. Beilby and Mott Jr. [4] develop a linear goal programming model for acquisition planning of academic libraries, and incorporate with multiple collection development goals such as acquiring an adequate number of titles (at least 7,500 but not more than 10,500 titles), not exceeding the total acquisition budget ($200,000), and/or limiting periodical expenditures to 60% of the total acquisition expenditures. Wise and Perushek [5] introduce another model that takes into account more goals, like reaching the minimum limit for each subject fund, not surpassing the maximum limit for each subject fund, and so forth. Later, Wise and Perushek [6] not only address an important claim that the suggestions of collection development librarians and faculties must be taken into consideration but also elaborate another model to reflect the opinion of librarians and faculties. Ho et al. [7] present a model that maximizes the average preference of patrons subject to both the acquisition cost and the number of materials in each category.

In most of the cases, academic libraries are positioned to acquire materials for multiple departments, for example, Science, Business, Engineering, and so forth, within the budget of each department. Goyal [8] proposes an operations research model of funds allocation to different departments of a university. The objective of this model is to maximize the total social benefits conveyed by the funds exercised for the purchase of materials among all departments, and the constraints of this model are the lower and upper limits of fund for each department and the total funds available. Arora and Klabjan [9] point out the critical concern about fairness in materials acquisition of academic libraries. They provide a model for maximizing the usage in the future time period subject to the bounds on the number of materials of each category and the lower and the upper bounds on the budgets of the library units. Existing researches on materials acquisition assume a single total budget or multiple department budgets. This study will investigate the scenario where each individual department has its own budget limit for the preferred materials that are to be acquired. This type of budget plan will introduce financial constraints that are much more complicated. 

From the viewpoint of acquisition staffs, it is questionable if the patrons are satisfied with the decision outcome. Niyonsenga and Bizimana [10] indicate various factors related to the patron satisfactions with academic libraries services, such as a list of new acquisitions, lending services, serial collection. In this paper, we adopt the patron preferences of acquisitions to reflect the patron satisfactions. To allocate the budget as fairly as possible, we assume that the preferences are obtained from the patrons of all departments due to the different interests of the departments. Besides, a low budget execution rate may lead to a budget cut in the next fiscal year. Librarians sometimes are on the horns of a dilemma whether to purchase the less preferred materials or cause a low budget execution rate. Therefore, we concentrate on how to select materials to be acquired in order to maximize the average preference as well as the budget execution rate under the real-world restrictions including departmental budget and limitation of the number of materials in each category.

In the view of computational complexity, the materials acquisition problem is a generalized version of the knapsack problem which is known to be computationally intractable [11]. In other words, it is extremely time consuming and even unlikely to find an optimal solution when the problem size is large. By far, metaheuristics, such as genetic algorithm, ant colony optimization, and particle swarm optimization are successfully applied to cope with many hard optimization problems with impressive performances in obtaining solutions with in an effective and efficient way [12, 13]. This paper is devoted to tackling the studied problem by particle swarm optimization (PSO) that has earned a good reputation by the trustworthy merits including simplicity, efficiency, and effectiveness in producing quality solutions [14, 15]. Furthermore, to avoid premature convergence, we introduce a discrete particle swarm optimization with *scout particles*, introduced by Silva et al. [16], to enhance the exploration capability of the adopted swarms.

The rest of this paper is organized as follows. In Section 2, a mathematical model of the materials acquisition problem with departmental demands is proposed and followed by a greedy algorithm.  Section 3 presents the fundamental concept and structure of the discrete particle swarm optimization (DPSO). In Section 4, we depict how the proposed DPSO with scout particles is tailored for the characteristics of the studied problem. A computational study is carried out to examine the performances of the proposed solution approaches. Our experimental settings and results of DPSO are presented in Section 5. We summarize the results of this study and give some concluding remarks in Section 6.

## 2. Problem Statements and Greedy Algorithm

A formal specification of the materials acquisition problem is presented in this section. Then, an integer programming model is developed to formulate the problem considered in a mathematical way.

### 2.1. Problem Specification

Consider a set of *n* materials to be acquired and a set of *m* departments. Each material is associated with a cost *c*_*i*_ and a preference value *p*_*ij*_ recommended by each department *j* for 1 ≤ *i* ≤ *n* and 1 ≤ *j* ≤ *m*. Each department owns an amount *B*_*j*_ of budget for 1 ≤ *j* ≤ *m*. Since one material may be recommended by more than one department, the acquisition cost would be apportioned by these recommending departments in proportion to their preferences. For instance, if a material with cost 100 is acquired to meet the recommendations from two departments *j* and *j*′ with preferences 0.9 and 0.6, then departments *j* and *j*′ should pay 40 ( = 100 × (0.9/(0.9 + 0.6))) and 60 ( = 100 × (0.6/(0.9 + 0.6))), respectively, from their budgets *B*_*j*_ and *B*_*j*′_. We denote the actual expense by department *j* for material *i* as *e*_*ij*_. To meet the acquisition requirements from various departments, *q* written languages (e.g., English, Japanese, Chinese, etc.) and *r* classified categories (e.g., Art, Science, Design, etc.) are considered such that the amount of materials belongs to a certain language and a specific category may be restricted into a range. In addition, the authority would expect the remainder of budget *B*_*j*_, once granted, for department *j* to be the less the better after allocation. We thus define the *execution rate* to be the actual expenses of all departments divided by the budget of all departments.

The decision is to determine which materials should be acquired and which departments should cover the cost associated with these materials under the constraints of departmental budgets and the limitation of the amounts in each written language and each category. The objective is to maximize the combination of the average preference and the budget execution rate.

In Table 1, we summarize the notations that will be used in the integer programming model throughout the paper.

### 2.2. Problem Formulation

The materials acquisition problem is mathematically formulated as the following integer programming model:

(1)  maximize O(x)=ρ×(∑j=1m(∑i=1nxijpij/∑i=1nxij)m)+(1−ρ)(∑i=1n∑j=1mxijeij∑j=1mBj)

(2) subject  to                 eij≥(xijpij∑j^=1mxij^pij^)×ci for  1≤i≤n,  1≤j≤m,

(3) $$\begin{array}{c} \sum_{i=1}^{n}e_{ij}\leq B_{j}\;\text{for}\;\;1\leq j\leq m, \end{array}$$

(4) $$\begin{array}{c} \sum_{i=1}^{n}x_{ij}-z_{i}M\leq 0\;\text{for}\;\;1\leq i\leq n, \end{array}$$

(5) $$\begin{array}{c} \sum_{i=1}^{n}x_{ij}+\left(1-z_{i}\right)M>0\;\text{for}\;\;1\leq i\leq n, \end{array}$$

(6) $$\begin{array}{c} \sum_{i=1}^{n}z_{i}a_{il}\leq LU_{l}^{}\;\text{for}\;\;1\leq l\leq r, \end{array}$$

(7) $$\begin{array}{c} \sum_{i=1}^{n}z_{i}a_{il}\geq LL_{l}^{}\;\text{for}\;\;1\leq l\leq r, \end{array}$$

(8) $$\begin{array}{c} \sum_{i=1}^{n}z_{i}b_{ik}\leq CU_{k}^{}\;\text{for}\;\;1\leq k\leq q, \end{array}$$

(9) $$\begin{array}{c} \sum_{i=1}^{n}z_{i}b_{ik}\geq CL_{k}^{}\;\text{for}\;\;1\leq k\leq q. \end{array}$$

The objective function (1) is to maximize the weighted sum of the average preference and the budget execution rate, where *ρ*, 0 ≤ *ρ* ≤ 1, is a parameter controlling the degree of importance between these two terms. The actual expense of material *i* apportioned by department *j* (*e*_*ij*_) is given in constraints (2), where all the cost of materials will be apportioned according to the proportion of the preference (*p*_*ij*_). Constraints (3) confine that the expense of any department *j* do not exceed its budget (*B*_*j*_). To ease the amount computation of the acquired materials, we introduce an auxiliary variable *z*_*i*_, which is 1 (0) if ∑_*j*=1_^*m*^ *x*_*ij*_ > 0 (otherwise), to show whether material *i* is acquired or not. Using a sufficiently large positive number *M*, constraints (4) and (5) are deliberately designed to obtain the proper value of *z*_*i*_. If ∑_*j*=1_^*m*^ *x*_*ij*_ ≤ 0, constraint (4) becomes irrelevant, where *z*_*i*_ may be either 0 or 1, but constraints (5) pledge *z*_*i*_ = 0, which indicates that material *i* is not acquired. On the contrary (∑_*j*=1_^*m*^ *x*_*ij*_ > 0), constraints (5) would be redundant, yet constraint (4) promises *z*_*i*_ = 1, which means that material *i* is acquired. If the material *i* is acquired (*z*_*i*_ = 1); then constraints (6) and (7) will force the number of acquired materials in each language *l* to be larger than or equal to the lower bounds and not to exceed the upper bounds. If material *i* is not acquired (*z*_*i*_ = 0), constraints (6) and (7) will assure the number of acquired materials in each language *l* included no material *i*. Constraints (11) and (12) are similarly defined to abide by the lower bound and upper bound specified on the number of materials in each category *k*.

### 2.3. Greedy Algorithm

A greedy solution method, denoted by Algorithm Greedy as shown in the Algorithm 1, is designed to be the comparison counterpart for other approaches. First, to decide if each material *i* will be acquired or not, all the materials are sorted in nonincreasing order of the ratio (∑_*j*=1_^*m*^ *p*_*ij*_)/*c*_*i*_. We thus assume the materials are reindexed in accordance with this sequencing rule. The first material, the one that attains the maximum (∑_*j*=1_^*m*^ *p*_*ij*_)/*c*_*i*_ ratio, will be considered if the following two conditions are satisfied: (1) the upper bound on the number of languages *LU*_*l*_ is not exceeded, and (2) the upper bound on the number of categories *CU*_*k*_ is not exceeded. Next, to determine which departments will apportion the cost of material *i*, all departments are sorted in nonincreasing order of *p*_*ij*_, and let (*j*_1_, *j*_2_,…, *j*_*m*_) be the sorted list. Material *i* will be acquired by department *j* (*x*_*ij*_ = 1), if the budget of this department is not exceeded.

## 3. Related Works of PSO

This section presents an overview on particle swarm optimization and describes two widely used topologies. What follows is a review on how to handle constraints and how to avoid premature convergence.

### 3.1. PSO

Particle swarm optimization (PSO) [14], introduced by Kennedy (a social psychologist) and Eberhart (an electrical engineer) in 1995 as an optimization method, is inspired by the observation on behavior of flocking birds and schooling fish. With the simplicity and lessened computation loads, PSO has been widely applied to many research areas, such as clustering and classification, communication networks, and scheduling [15, 17–19].

In foraging, birds flock together and arrange themselves in specific shapes or formations by sharing their information about food sources. The movement of each particle will be influenced by the experiences of itself and the peers. In the process of optimization, each particle *s* of flock *S* is associated with a *position*, a *velocity*, and a *fitness value*. A position, which is a vector in a search space, represents a potential solution to an optimization problem; a velocity, which is a vector, represents a change in the position; a fitness value, which is computed by the objective function, indicates how well the particle solves the problem. 

To find an approximate solution, each particle *s* determines its movement iteratively by learning from its own experience and communication with its neighbors. The mechanism of coordination is encapsulated by the *velocity control* over all particles at each iteration *t* of the algorithm. For each particle *s*, the velocity at iteration *t* + 1 (*V*_*s*_^*t*+1^) is updated with (10), where *P*_*s*_^*t*^ denotes the solution found by (position of) particle *s* at iteration *t*, ${\overset{-}{P}}_{s}^{t}$ denotes the best solution found by particle *s* until iteration *t*, and ${\hat{P}}_{s}^{t}$ denotes the best solution found by the neighbors of particle *s*. The *cognition learning* rate (*c*_1_) and *social learning* rate (*c*_2_) are introduced to control the influence of individual experience and their neighbors' experience, respectively. At the next iteration *t* + 1, the position of each particle is updated by (11). One has

(10) $$\begin{array}{c} V_{s}^{t+1}=V_{s}^{t}+c_{1}r_{1}\left({\overset{-}{P}}_{s}^{t}-P_{s}^{t}\right)+c_{2}r_{2}\left({\hat{P}}_{s}^{t}-P_{s}^{t}\right), \end{array}$$

(11) $$\begin{array}{c} P_{s}^{t+1}=P_{s}^{t}+V_{s}^{t+1}. \end{array}$$

For discrete optimization problems, Kennedy and Eberhart [20] also introduce a binary particle swarm optimization that changes the concept of velocity from adjustment of the position to the probability that determines whether a bit of a solution becomes one or zero. The velocity of each particle *s* at iteration *t*, *V*_*s*_^*t*+1^, is squashed in sigmoidal function as shown in (12); the position updating function is replaced by (13), where rand() is a random number drawn from the interval [0, 1]. One has

(12) $$\begin{array}{c} S\left(V_{s}^{t+1}\right)=\frac{1}{1+e^{-(V_{s}^{t+1})}}, \end{array}$$

(13) $$\begin{array}{c} P_{s}^{t+1}=\{\begin{array}{cc} 1 & \text{if}\;\;\text{rand}\left(\right)<S\left(V_{s}^{t+1}\right),\\ 0 & \text{otherwise}, \end{array} \end{array}$$

To better balance the exploration and exploitation, several variants of PSO algorithm have been proposed in the literature. A widely used method, proposed by Eberhart and Shi [21], is to introduce an *inertia weight* (*w*) to the velocity updating function shown in (14). The inertia weight is used to adjust the influence of the current velocity on the new velocity:

(14) $$\begin{array}{c} V_{s}^{t+1}=wV_{s}^{t}+c_{1}r_{1}\left({\overset{-}{P}}_{s}^{t}-P_{s}^{t}\right)+c_{2}r_{2}\left({\hat{P}}_{s}^{t}-P_{s}^{t}\right). \end{array}$$

### 3.2. Communication Topology

In the literature, several communication topologies have been extensively studied. Poli et al. [22] classify the communication structures into two categories: static topologies and dynamic topologies. Static topologies are that the number of neighbors does not change at all iterations of a run; dynamic topologies, on the other hand, are that the size of neighborhoods dynamically increases. 

Local topology, global topology, and von Neumann topology are some well-known examples of static topology. As for dynamic topologies, the neighborhood size can be influenced by a dynamic hierarchy, a fitness distance ratio, or a randomized connection, just to name a few. The canonical PSO algorithm, proposed by Bratton and Kennedy [23], is equipped with global and local topologies.

A PSO with a global topology (or *gbest* topology) allows each particle to communicate with all other particles in the swarm, while a PSO with a local topology (or *lbest* topology) allows each particle to share information with only two other particles in the swarm. Therefore, a *gbest* PSO could lead to a faster convergence but might be trapped into a local optimal solution. Conversely, an *lbest* PSO could result in a slower rate of convergence but might be able to escape from a local optimal.

### 3.3. Constraint Handling

As reported in the literature, there are various different methods for handling constrained optimization problems. Several commonly used methods are based on penalty functions, rejection of infeasible solutions, repair algorithm, specialized operators, and behavioral memory [24–26]. In this paper, we focus on the method based on penalty function. Details concerning the penalty function for the studied problem are given in the next section.

When implementing penalty functions, the fitness evaluation for a solution is not just dependent on the objective function but incorporated the penalty function with the objective function. This method can be implemented as stationary or nonstationary. If there is an infeasible solution, the stationary penalty function simply adds a fixed penalty. Contrary to the stationary one, the nonstationary function adds a floating penalty which changes the penalty value according to the violated constrains and the iterations number. Parsopoulos and Vrahatis [25] note that the results obtained by nonstationary penalty functions are superior to the stationary one for the most of the time. A high penalty leads to a feasible solution even it is not approximate to the optimal solution, while a low penalty reduces the probability to obtain a feasible solution. Therefore, Coath and Halgamuge [24] point out that a fine-tuning of the parameters in the penalty function is necessary when using this method. The method based on the rejection of infeasible solution is to discard an infeasible solution even if it is closer to the optimal solution than some feasible ones. The repair algorithm, an extensively employed method in genetic algorithms (GA), is equipped to fix an infeasible solution, but the cost is more computationally expensive than other methods.

### 3.4. Avoiding Premature Convergence

Most of the global optimization methods suffer from premature convergence. One of the most used approaches to tackle this problem is to introduce diversity to the velocity or the position of a particle. As mutation operators are to the genetic algorithm, so is introduction of diversity to PSO algorithms. The focus of this paper is to introduce the diversity by employing scout particles. The details of how the proposed DPSO algorithm circumvents premature convergence are described in Section 4.

García-Villoria and Pastor [27] introduce the concept of diversity into the velocity updating function. The proposed dynamic diversity PSO (PSO-*c3dyn*) dynamically changes the diversity coefficients of all particles through iterations. The more heterogeneity of the population will be, the less diversity will be introduced to the velocity updating function, and vice versa. Blackwell and Bentley [28] incorporate diversity into the population by preventing the homogeneous particles from clustering tightly to each other in the search space. They provide collision-avoiding swarms that reduce the attraction of the swarm center and increase the coverage of a swarm in the search space. Silva et al. [16] attempt to apply the diversity to both the velocity and the population by a predator particle and several scout particles. A predator particle is intended to balance the exploitation and exploration of the swarm, while scout particles are designed to implement different exploration strategies. The closer the predator particle will be to the best particle, the higher probability of perturbation will be.

## 4. DPSO with Scout Particles

This section details how to tackle the materials acquisition problem by discrete particle swarm optimization with scout particles. The representation of a particle and the initialization method for the studied problem are described in Section 4.1. Then, Section 4.2 elaborates on the details of preventing premature convergence by deploying scout particles.  Section 4.3 redefines a constraints handling mechanism for solving the constrained optimization problem.

### 4.1. Representation and Initialization

The solution of materials acquisition problem with *n* materials and *m* departments obtained by particle *s* at iteration *t* can be represented by an *n* × *m* binary matrix, proposed by Wu et al. [29], as shown in (15). Each entry of the matrix (*P*_*s*_^*t*^)_*ij*_ indicates whether material *i* is acquired by department *j* or not. Note that each entry of the matrix (*P*_*s*_^*t*^)_*ij*_ corresponds to the decision variable (*x*_*ij*_) that was mentioned in Section 2.1:

(15) $$\begin{array}{c} P_{s}^{t}=\left[\begin{array}{ccc} P_{s(11)}^{t} & \cdots & P_{s(1m)}^{t}\\ \vdots & \ddots & \vdots \\ P_{s(n1)}^{t} & \cdots & P_{s(nm)}^{t} \end{array}\right]. \end{array}$$

The initial population is generated by setting a void velocity and randomly generated entries of matrix *P*_*s*_^*t*^ for each particle *s*. To find feasible solutions for the initial population, an initialization procedure is designed and depicted in Algorithm 2. To determine which constraint should be satisfied first, the sum of the lower bounds on the numbers of materials in all languages ∑_*l*=1_^*r*^ *LL*_*l*_ and in all categories ∑_*k*=1_^*q*^ *CL*_*k*_ are computed. The one with less sum will be satisfied first by randomly selecting the material that belongs to language *l* or category *k*.

### 4.2. Constraints Handling

In the literature, repair operators and penalty functions are widely used approaches to handling constrained optimization problems. However, due to the computationally heavy load of repair operators, we focus on solely penalty functions. For each particle, the fitness value is evaluated by (16), where *O*(*x*_*ij*_) is the objective value of the studied problem given in (1), and *H*(*x*_*ij*_) is a penalty factor defined in (17). A feasible solution reflects its objective value as the fitness value, while an infeasible solution receives an objective value and a penalized value by (17). It can be seen from (17) that each term is associated with constrains (3), (6), (7), (8), and (9), as mentioned in Section 2.2. For instance, if a solution reports that the expense of any department *j* exceeds the budget *B*_*j*_, addressed in constraints (3), then a positive penalty value can be subtracted from the fitness value to reflect the infeasibility. One has

(16) $$\begin{array}{c} F\left(x_{ij}\right)=O\left(x_{ij}\right)-H\left(x_{ij}\right), \end{array}$$

(17) H(xij)=∑j=1mmax{0,∑i=1nxijeij−BjBj}+∑l=1rmax{0,∑i=1nyiail−LUl|∑i=1nyiail−LLl|}+∑l=1rmax{0,LLl−∑i=1nyiail|LUl−∑i=1nyiail|}+∑k=1qmax{0,∑i=1nyibik−UCk|∑i=1nyibik−CLk|}+∑k=1qmax{0,CLk−∑i=1nyibik|CUk−∑i=1nyibik|}.

### 4.3. Scout Particles

Premature convergence is a challenging problem faced by PSO algorithms throughout the optimization process. To avoid premature convergence in the DPSO algorithm for the studied problem, this paper employs scout particles to enhance the exploration. The concept is to send out scout particles to explore the search space and collect more extensive information of optimal solutions for other particles. If a scout particle finds a solution that is quite different from the best solution and the expected fitness value is better, the scout particle will share the information with some particles by affecting their velocities.

**Figure 1:**
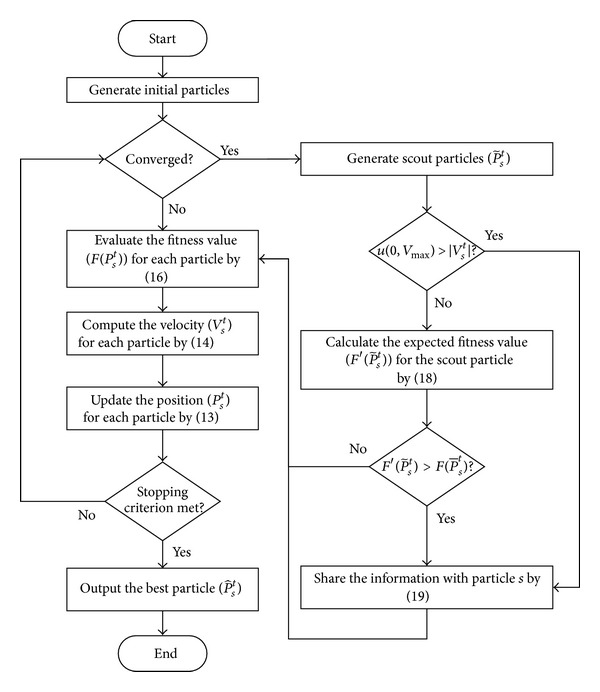
DPSO with scout particles.

The DPSO procedure with scout particles is depicted in Figure 1. Firstly, in order to generate a feasible swarm, the particles are generated by the initialization procedure as mentioned in Section 4.1. Secondly, when the swarm has not yet converged, the regular particle *s* (*P*_*s*_^*t*^) flies through the search space by the following steps: fitness evaluation, velocity calculation, and position updating. If the swarm converges, on the other hand, scout particles ${\tilde{P}}_{s}^{t}$ will be generated for exploration by randomly selecting a material to be acquired by all departments until the solution meets the lower bound and the upper bound on the number of languages and categories. In this paper, the convergence of DPSO is specified by the fitness variance. 

The scout particles will share the information with the peer particles subject to a probability that depends on the velocity of each particle *s*. The larger velocity of particle *s*, the higher probability of the scout particle affecting the particle *s* by (19), where the diversity coefficient (*c*_3_) is a prespecified parameter and *r*_3_ is a random number drawn from the interval [0, 1]. Also, if the expected fitness value of the scout particle ${\tilde{P}}_{s}^{t}$ is greater than the fitness of the best solution bound by particle ${\overset{-}{P}}_{s}^{t}$, the particles will share information with other particles by (19). The expected fitness of the scout particle ${\tilde{P}}_{s}^{t}$ is calculated by (18), where *ρ* is a nonnegative weight and *p*_*i*_ is the total preference of material *i* cast by all departments, *p*_*i*_ = ∑_*j*=1_^*m*^ *p*_*ij*_. One has

(18) F′(P~st)=ρ×∑i=1n(P~st)pi/∑i=1n(P~st)m+(1−ρ)×∑i=1n(P~st)ci∑j=1mBj,

(19) $$\begin{array}{c} V_{s}^{t+1}=wV_{s}^{t}+c_{3}r_{3}\left({\tilde{P}}_{s}^{t}-P_{s}^{t}\right). \end{array}$$

## 5. Computational Experiments

To manifest the effectiveness and efficiency of the proposed DPSO of materials acquisition, a series of computational experiments were designed and conducted. The experiment setting and test instances are described in Section 5.1 and the computational results and analysis are given in Section 5.2.

### 5.1. Test Instances and Settings

Small-size test instances and large-size test instances are exhibited in Tables 2 and 3, respectively. The number of materials *n*, the number of departments *m*, the budget limits *B*_*j*_ of department *j*, the number of languages *r*, the lower bound on the number of materials *LL*_*l*_ in language *l*, the upper bound on the number of materials *LU*_*l*_ in language *l*, the number of categories *q*, the lower bound on the number of materials *CL*_*k*_ in category *k*, the upper bound on the number of materials *CU*_*k*_ in category *k* were tabulated. The small-size test instances (Case I), determined by the combinations of *n*, *m*, *r*, and *q*, were composed of 60 ( = 3 × 5 × 2 × 2) instances. The large-size test instances (Case II) were composed of 20 ( = 5 × 2 × 2) instances, where *n* was 100,000.

The default values of the parameters in both DPSO and DPSO with scout particles algorithms were set as particle size *S* = 30, number of iterations *t* = 500, inertia weight *w* = 0.9, cognition learning rate *c*_1_ = 2.05, social learning rate *c*_2_ = 2.05, and diversity coefficient *c*_3_ = 0.5. The number of scout particles was set to one. All of the programs were implemented in C#.net and run on a PC with an Intel Core i5-2400 3.1 GHz CPU and 4 G RAM. The stopping criteria of all test cases were defined as no improvement on the incumbent solution can be achieved within 50 consecutive iterations. 

### 5.2. Results and Analysis

To understand the effectiveness and efficiency of the proposed DPSO, we examine the four key features, including initialization, swarm topology, constraints handling, and scout particles. The following subsections detail the results and analysis (Tables 4–7). The rows labeled “*Average*” and “*Stdev*” in each table list the average and standard deviations of improvement and execution time for several observations. The next three rows in each table report the number of observations on the results of different DPSO algorithms for the test instances, the *z*-score of statistical test where the null hypothesis is that the different features of DPSO algorithm have the same improvement (or execution time), and the *P* value which is translated from *z*-score. Note that the number of observations for case I (resp., II) is set as 480 (resp., 160), the combinations 8 ( = 2 × 2 × 2) of features for 60 (resp., 20), for the purpose of evading the influence of other features. The significance level *α* is set at 0.05. Also, to facilitate a comparison of the effectiveness of the proposed DPSO algorithm across different test instances, the improvement in percentage over Algorithm Greedy, calculated as in (20), is employed instead of an absolute difference in objective value:

(20) $$\begin{array}{c} \text{improvement}=\left(\frac{\text{DPSO}-\text{greedy}}{\text{greedy}}\right)\%. \end{array}$$

#### 5.2.1. Initialization

Results of different initialization strategies on the 60 small-size test instances (Case I) and 20 large-size test instances (Case II) are summarized in Table 4. The column labeled “*Random*” reports the results of DPSO algorithm that generates the initial swarms by the proposed initialization procedure in Section 4.1; the column labeled “*Greedy*” reports the results of DPSO algorithm that generates the initial swarms by both the abovementioned initialization procedure and the Algorithm Greedy in Section 2.3. 

It can be seen from Table 4 that the improvements achieved by two different initialization strategies are appealing. For case I, the improvement on the random strategy is slightly better than that on the greedy strategy (52.46% versus 52.11%); for case II, the greedy strategy performs slightly better (73.01% versus 71.32%). However, the difference in improvement between the “Random” and “Greedy” initializations for case I and case II yielded *P* values of 0.8460 and 0.6825 using *z*-test at *α* of 0.05. Therefore, the difference in improvement of two initialization strategies is not statistically significant. We could thus reason that the DPSO equipped with these different initialization strategies will lead to the same significant improvement rate.

Regarding the execution time, both initialization strategies can produce solution for small test instances (Case I) in a very short time. The difference in execution time between the “*Random*” and “*Greedy*” initialization on case I and II yielded a *P* value of 0.5918 and 0.5590 by *z*-test at *α* = 0.05. It reveals that the difference is not statistically significant on both cases. This phenomenon is reasonable because both of the initialization strategies enable the diversity of initial swarms before they satisfy the stopping criterion. These results suggest that DPSO can obtain good solutions with these initialization strategies.

#### 5.2.2. Swarm Topology

Results of different swarm topologies on the 60 small-size test instances (Case I) and 20 large-size test instances (Case II) are summarized in Table 5. The columns labeled “*Star*” and “*Ring*” list the results of DPSO algorithm with star topology and ring topology.

From Table 5, the improvements of both star and ring topologies on two test cases reached a high percentage (on average 62.25%), being quite attractive. For cases I and II, the difference in execution time yielded a *P* value less than 0.05 (*P* value = 0.0000), indicating that a statistically significant difference in improvement existed. Accordingly, we would suggest that star topology (*gbest*) is an effective swarm topology to deliver solutions with satisfactory qualities.

In Table 5, the results of execution time needed by different topologies reaffirm the fact that star topology (*gbest*) seem to have a faster convergence rate than the ring topology (*lbest*). For small-size test instances (Case I), the *z*-test of the difference in execution time between star topology (1.31 seconds) and ring topology (1.92 seconds) yielded a *P* value less than 0.05, indicating that a statistically significant difference in execution time exists; for large-size test instances (Case II), even though the star topology spent less computation time, the difference in execution time between the star topology (772.26 seconds) and ring topology (808.72 seconds) yielded a *P* value of 0.3076 by *z*-test at *α* = 0.05, specifying that no statistically significant difference in execution time was found. This is reasonable because of the large standard deviation in the results of case II. The result suggests that the star topology spent less computational time to obtain attractive solutions to the studied problem.

#### 5.2.3. Constraints Handling

Results of different constraints handling mechanisms on Cases I and II are shown in Table 6. The column labeled “*Accept*” reveals the results of DPSO algorithm that accept infeasible solutions as the best solution found by particle *s* at iteration *t* (${\overset{-}{P}}_{s}^{t}$); on the other hand, the column labeled “*Reject*” reveals those reject infeasible solutions. 

As can be seen from Table 6, the improvements of two different constraint handling approaches do produce good solutions. For the small-size test instances (Case I), the average improvements of the “Accept” mechanism and the “Reject” mechanism are 52.81% and 51.76%; for the large-size test instances (Case II), the average improvements are 72.85% and 71.48%. The results show that the “Accept” mechanism reaches slightly higher improvement than the “Reject” mechanism in both cases within a longer execution time. This is reasonable because the “Accept” mechanism has more chance to explore the infeasible solution space and takes more iteration to converge. However, to have a concise comparison of “Reject” mechanism and the “Accept” mechanism, the *z*-test yields *P* values of 0.5603 and 0.7410, which indicate that there is no statistical difference. The computational results and analysis shown in Table 6 suggest that DPSO with both constraints handling mechanisms can produce quality solutions.

#### 5.2.4. Scout Particles

Results of DPSO and DPSO with scout particles on two test instances (small size and large size) are exhibited in Table 7. The column labeled “*Scout*” displays the results of DPSO algorithm with scout particles, while the column labeled “*Standard*” displays the results of DPSO algorithm without scouts.

For the improvement, the DPSO with scouts does produce better solutions than the standard DPSO on all test instances. It can be seen from Table 7 that the DPSO with scout particles reported 57.21% improvement rate on small-size test instances and 81.34% improvement rate on large-size test instances, while the standard DPSO showed 47.36% and 62.99%. The *z*-test of the difference in improvement yielded a *P* value less than 0.05 which indicates that a statistically significant difference in execution time existed. The effectiveness of the proposed DPSO can be attributed to the scout particles that decrease the chance to be trapped in local optimal by exploring the search space. This reveals that the proposed DPSO is an effective approach to the problem.

As for the execution time, the DPSO with scouts took less computation time than the standard DPSO on all test instances as well. In Table 7, the DPSO with scout particles took 1.23 seconds for solving the small-size test instances and 741 seconds 741 for large-size test instances. On the other hand, the elapsed times of the standard DPSO are 2.01 seconds and 839.97 seconds. For each case, the *z*-test yields a *P* value below 0.05, indicating that the difference in execution times is significant. This result evinces the efficiency of the DPSO with scouts by showing that the time elapsed is smaller than the standard DPSO. This phenomenon may be due to the fact that scout particles were evaluated by the expected fitness instead of the objective function. 

## 6. Conclusions

In this paper, we have proposed an integer programming model for the materials acquisition problem, which is to maximize both the average preference and the budget execution rate being subject to some constraints of the budget, the required number of materials in each category and language. To solve the constrained problem, we have developed a DPSO algorithm and designed an initialization strategy to generate feasible particles. We have also conducted computational experiments of two sets test instances to demonstrate the effectiveness and efficiency of the proposed DPSO algorithm.

To better solve the studied problem, four different features of the proposed DPSO, including initialization strategies, swarm topology, constraints handling mechanism, and scout particles, are discussed. Firstly, we compare the results of employing the proposed initialization procedure, and the results of employing both the proposed Algorithm Greedy and initialization procedure. The computational results show that DPSO algorithm can obtain quality solutions with both the initialization strategies in a reasonable time. Secondly, we compare the results of performing star topology and ring topology. The results evince that star topology significantly outperforms ring topology in all test instances. Next, we compare the performances resulted from different constraint handling mechanisms. One mechanism is to accept the infeasible solutions as the best solution found by each particle, while the other is to reject the infeasible solutions as the best solution found by each particle. The computational results demonstrate that these two mechanisms reach the same performance. Lastly, we compare the results of standard DPSO and DPSO with the proposed scout particles. The results reveal that DPSO with scouts reaches higher improvement rates and takes shorter execution time. Accordingly, we would suggest that DPSO with the proposed initialization procedure, star topology, and scout particles is an effective approach to delivering attractive solutions in a reasonable time.

## Figures and Tables

**Algorithm 1:**
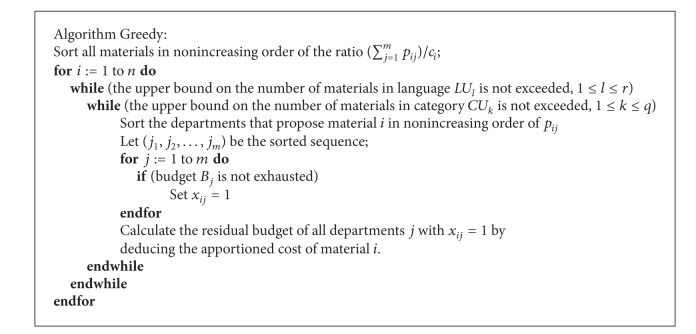
Greedy solution method.

**Algorithm 2:**
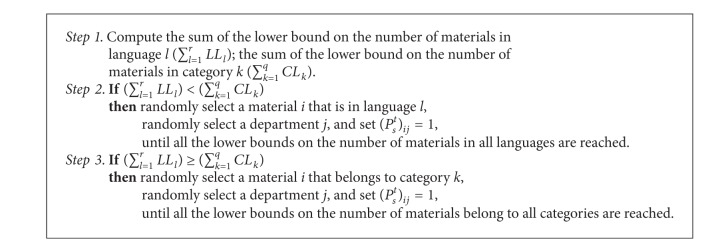
Initialization procedure of DPSO.

**Table 1:** Notations.

Variable	Description
*n*	Number of materials
*m*	Number of departments
*q*	Number of categories
*r*	Number of languages
*p*_*ij*_	Preference for material *i* recommended by department *j*, for 1 ≤ *i* ≤ *n* and 1 ≤ *j* ≤ *m*
*c*_*i*_	Cost of material *i*, for 1 ≤ *i* ≤ *n*
*B*_*j*_	Budget limit of department *j*, for 1 ≤ *j* ≤ *m*
*LU*_*l*_	Upper bound on the number of materials in language *l*, for 1 ≤ *l* ≤ *r*
*LL*_*l*_	Lower bound on the number of materials in language *l*, for 1 ≤ *l* ≤ *r*
*a*_*il*_	*a*_*il*_ = 1 if material *i* is in language *q*; *a*_*il*_ = 0 otherwise, for 1 ≤ *i* ≤ *n* and 1 ≤ *l* ≤ *r*
*CU*_*k*_	Upper bound on the number of materials in category *k*, for 1 ≤ k ≤ q
*CL*_*k*_	Lower bound on the number of materials in category *k*, for 1 ≤ k ≤ q
*b*_*ik*_	*b*_*ik*_ = 1 if material *i* belongs to category *k*; *b*_*ik*_ = 0 otherwise, for 1 ≤ *i* ≤ *n* and 1 ≤ *l* ≤ *r*
*x*_*ij*_	Decision variable: *x*_*ij*_ = 1 if material *i* is acquired for department *j* from which the cost will be charged; *x*_*ij*_ = 0 otherwise, for 1 ≤ *i* ≤ *n* and 1 ≤ *j* ≤ *m*
*z*_*i*_	Auxiliary variable: *z*_*i*_ = 1, if ∑_*j*=1_^*m*^ *x*_*ij*_ > 0; otherwise *z*_*i*_ = 0, for 1 ≤ *i* ≤ *n* (*z*_*i*_ reveals whether material *i* is acquired or not)
*e*_*ij*_	Actual expenses of material *i* by department *j*, for 1 ≤ *i* ≤ *n* and 1 ≤ *j* ≤ *m*

**Table 2:** Small-size test instances, Case I.

*n*	{*m*, {*B*_*j*_}}	{*r*, {*LU*_*l*_}, {*LL*_*l*_}}	{*q*, {*CU*_*k*_}, {*CL*_*k*_}}
100	{1, {15000}}, {2, {6000, 9000}}, {3, {3000, 3000, 4000}}, {4, {3000, 3000, 4500, 4500}}, {5, {1500, 1500, 3000, 4500, 4500}}.	{2, {10, 20}, {5, 12}}, {3, {5, 10, 15}, {3, 3, 3}}.	{3, {6, 6, 12}, {3, 3, 6}}, {5, {3, 3, 6, 9, 9}, {3, 3, 3, 3, 3}}.

200	{1, {20000}}, {2, {8000,12000}}, {3, {4000, 10000, 6000}}, {4, {3000, 3000, 3000,4000, }}, {5, {2000, 2000, 2000, 8000, 4000, }}.	{2, {15, 25}, {5, 10, }}, {3, {10, 15, 15}, {5, 5, 5}}.	{3, {10, 10, 20}, {2, 4, 6}}, {5, {4, 4, 8, 12, 12}, {0, 0, 2, 3, 3}}.

300	{1, {30000}}, {2, {10000,20000}}, {3, {6000, 6000, 18000, }}, {4, {6000, 6000, 9000, 9000}}, {5, {2000, 4000, 7000, 8000, 9000}}.	{2, {25, 35}, {10, 20}}, {3, {15, 15, 30}, {10, 10, 15}}.	{3, {10, 20, 30}, {5, 10,10}}, {5, {10, 10, 10, 15, 15}, {5, 5, 5, 5, 5}}.

**Table 3:** Large-size test instances, Case II.

{*m*, {*B*_*j*_}} (unit of *B*_*j*_: 10000)	{*r*, {*LU*_*l*_}, {*LL*_*l*_}}	{*q*, {*CU*_*k*_}, {*CL*_*k*_}}
{5, {80, 80, 100, 120, 120}}, {10, {40, 40, 40, 50,50, 50, 50, 60, 60, 60}}, {15, {30, 30, 30, 30, 30, 33, 33, 33, 33, 33, 36, 36, 36, 36, 36}}, {20, {20, 20, 20, 20, 20, 25, 25, 25, 25, 25, 25, 25, 25, 25, 25, 30, 30, 30, 30}}, {25, {16, 16, 16, 16, 16, 18, 18, 18, 18, 18, 20, 20, 20, 20, 20, 22, 22, 22, 22, 22, 24, 24, 24, 24, 24}}.	{2, {4000, 6000}, {1000, 2000}}, {3, {3000, 3000, 4000}, {500, 500, 1000}}.	{5, {1000, 1000, 2000, 3000, 3000}, {200, 400, 800, 1000, 1200}}, {10, {600, 600, 800, 1000, 1000, 1000, 1200, 1400, 1400}, {100, 200, 300, 500, 500, 600, 700, 700, 800, 1000}}.

**Table 4:** Results of different initialization strategies on two test cases.

Case	Measure	Improvement		Execution time	
		Random	Greedy	Random	Greedy
I	Average	52.46%	52.11%	1.6455	1.5956
	Stdev	0.2805	0.2795	1.5258	1.3455
	Observations	480	480	480	480
	*z*-score	0.1942		0.5362	
	*P* value	0.8460		0.5918	

II	Average	71.32%	73.01%	779.9824	800.9922
	Stdev	0.3675	0.3722	318.31	324.77
	Observations	160	160	160	160
	*z*-score	−0.4090		−0.5843	
	*P* value	0.6825		0.5590	

**Table 5:** Results of different swarm topologies on two test cases.

Case	Measure	Improvement		Execution time	
		Star	Ring	Star	Ring
I	Average	62.47%	42.10%	1.3193	1.9219
	Stdev	0.2244	0.2216	0.4285	1.9427
	Observations	480	480	480	480
	*z*-score	12.1029		−6.6364	
	*P* value	0.0000		0.0000	

II	Average	80.98%	63.35%	772.2568	808.7178
	Stdev	0.4147	0.2934	370.76	262.47
	Observations	160	160	160	160
	*z*-score	4.3889		−1.0202	
	*P* value	0.0000		0.3076	

**Table 6:** Results of different constraints handlings on two test cases.

Case	Measure	Improvement		Execution time	
		Accept	Reject	Accept	Reject
I	Average	52.81%	51.76%	1.6610	1.5802
	Stdev	0.2826	0.2773	1.4622	1.4135
	Observations	480	480	480	480
	*z*-score	0.5825		0.8703	
	*P* value	0.5603		0.3841	

II	Average	72.85%	71.48%	803.15	777.82
	Stdev	0.3693	0.3705	310.83	331.79
	Observations	160	160	160	160
	*z*-score	0.3304		0.7047	
	*P* value	0.7410		0.4810	

**Table 7:** Results of DPSO with and without scout particles on two test cases.

Case	Measure	Improvement		Execution time	
		Standard	Scout	Standard	Scout
I	Average	47.36%	57.21%	2.0065	1.2346
	Stdev	0.2443	0.3037	1.8070	0.7588
	Observations	480	480	480	480
	*z*-score	−5.5393		8.6285	
	*P* value	0.0000		0.0000	

II	Average	62.99%	81.34%	839.9745	741.0001
	Stdev	0.3015	0.4073	295.65	338.65
	Observations	160	160	160	160
	*z*-score	−4.5795		2.7849	
	*P* value	0.0000		0.0054	
